# Clinical Decision Support System Used in Spinal Disorders: Scoping Review

**DOI:** 10.2196/53951

**Published:** 2024-03-19

**Authors:** Zheng An Toh, Bjørnar Berg, Qin Yun Claudia Han, Hwee Weng Dennis Hey, Minna Pikkarainen, Margreth Grotle, Hong-Gu He

**Affiliations:** 1 National University Hospital National University Health System Singapore Singapore; 2 Centre for Intelligent Musculoskeletal Health Faculty of Health Sciences Oslo Metropolitan University Oslo Norway; 3 Department of Nursing Tan Tock Seng Hospital Singapore Singapore; 4 Division of Orthopaedic Surgery National University Hospital National University Health System Singapore Singapore; 5 Yong Loo Lin School of Medicine National University of Singapore Singapore Singapore; 6 Department of Rehabilitation and Health Technology Oslo Metropolitan University Oslo Norway; 7 Martti Ahtisaari Institute, Oulu Business School Oulu University Oulu Finland; 8 Department of Product Design Oslo Metropolitan University Oslo Norway; 9 Department of Research and Innovation Division of Clinical Neuroscience Oslo University Hospital Oslo Norway; 10 Alice Lee Centre for Nursing Studies Yong Loo Lin School of Medicine National University of Singapore Singapore Singapore

**Keywords:** back pain, clinical decision support systems, CDSS, diagnosis, imaging, predictive, prognosis, spine

## Abstract

**Background:**

Spinal disorders are highly prevalent worldwide with high socioeconomic costs. This cost is associated with the demand for treatment and productivity loss, prompting the exploration of technologies to improve patient outcomes. Clinical decision support systems (CDSSs) are computerized systems that are increasingly used to facilitate safe and efficient health care. Their applications range in depth and can be found across health care specialties.

**Objective:**

This scoping review aims to explore the use of CDSSs in patients with spinal disorders.

**Methods:**

We used the Joanna Briggs Institute methodological guidance for this scoping review and reported according to the PRISMA-ScR (Preferred Reporting Items for Systematic Reviews and Meta-Analyses Extension for Scoping Reviews) statement. Databases, including PubMed, Embase, Cochrane, CINAHL, Web of Science, Scopus, ProQuest, and PsycINFO, were searched from inception until October 11, 2022. The included studies examined the use of digitalized CDSSs in patients with spinal disorders.

**Results:**

A total of 4 major CDSS functions were identified from 31 studies: preventing unnecessary imaging (n=8, 26%), aiding diagnosis (n=6, 19%), aiding prognosis (n=11, 35%), and recommending treatment options (n=6, 20%). Most studies used the knowledge-based system. Logistic regression was the most commonly used method, followed by decision tree algorithms. The use of CDSSs to aid in the management of spinal disorders was generally accepted over the threat to physicians’ clinical decision-making autonomy.

**Conclusions:**

Although the effectiveness was frequently evaluated by examining the agreement between the decisions made by the CDSSs and the health care providers, comparing the CDSS recommendations with actual clinical outcomes would be preferable. In addition, future studies on CDSS development should focus on system integration, considering end user’s needs and preferences, and external validation and impact studies to assess effectiveness and generalizability.

**Trial Registration:**

OSF Registries osf.io/dyz3f; https://osf.io/dyz3f

## Introduction

### Background

Spinal diseases are a group of conditions that affect the spinal column, leading to various symptoms ranging from pain to paralysis. The types of conditions may include spinal stenosis, herniated disc, scoliosis, osteoporosis, and degenerative disc disease, each with a unique etiology [1]. These conditions can be caused by various factors, such as genetic predisposition; age-related degeneration; trauma; infections; autoimmune and metabolic disorders; and lifestyle choices, including posture, exercise, and weight management [2]. Low back pain (LBP) is a significant health problem highly associated with spinal disorders [2], which affected an estimated 7.5% of the world’s population in 2017, with approximately 568.4 million cases reported worldwide in 2019 [3]. It has prevailed as the leading cause of disability worldwide, contributing to 63.7 million years lived with disability as of 2019, influencing people of working age (from 20 to 65 years) and beyond [4]. In 2017, the cost of LBP topped the health care spending in the United States, estimated at US $134.5 billion [5]. Furthermore, LBP leads to wage and productivity losses, reflecting high costs to society [6-8]. Consequently, significant research efforts have been placed on spinal disorders, including technological patient management.

Presently, physicians are encouraged to deploy an evidence-based approach toward diagnosis and treatment by considering the best scientific (ie, matching symptoms and signs with relevant investigations and ensuring that the radiological features are concordant with the observed symptoms and signs) or research evidence and clinical experience while considering patients’ values and preferences [9]. However, the overwhelming number of scientific publications makes it challenging for physicians to stay updated with the latest evidence. To address this issue, computer-based tools, such as clinical decision support systems (CDSSs), can be used.

CDSSs are computerized tools used in health care to provide personalized treatment recommendations, aid in clinical diagnosis, and predict patient-specific outcomes and prognoses [10]. These tools significantly enhance disease management in health care by improving diagnostic accuracy through timely information and narrowing down potential conditions [10]. It ensures that evidence-based treatment recommendations align with current medical guidelines, aiding medication management with alerts for interactions and allergies [11]. In personalized medicine, CDSSs use genetic data for tailored treatment plans [10]. They allow the optimization of health care workflows, reduces errors, and improves communication among professionals, thereby enhancing patient outcomes and efficient health care delivery [11]. The CDSSs can be broadly classified into knowledge-based and non–knowledge-based systems. Knowledge-based CDSSs use rules to match patient data with preset knowledge domains based on up-to-date, evidence-based clinical information, from which the best recommendations can be derived [11]. In contrast, non–knowledge-based systems use data-driven methods such as artificial intelligence (AI) or machine learning to make predictions or decisions. Although limited by their lack of transparency and auditing capability, non–knowledge-based systems can provide alternative perspectives and highlight potentially overlooked factors [10]. Recently, newer methods have been developed to interpret some AI findings, offering the possibility of greater acceptance of the non–knowledge-based methodology [12,13].

A systematic review and meta-analysis reported a 10% to 20% decrease in morbidity when CDSSs were used in patient care [14]. Physicians using CDSSs are more likely to order appropriate treatment or therapy and make fewer medication errors, thereby improving overall patient safety [10,15]. Despite these successes, research regarding the use of CDSSs in spinal disorders is still in its infancy, with much to be explored.

### Objectives

Previous reviews have investigated the diagnostic and predictive performances of AI and machine learning [16-26]. However, no systematic or scoping review on the use of CDSSs in patients with spinal disorders has been identified. Therefore, this scoping review aimed to assess the extent of the literature in which CDSSs were implemented in clinical practice to assist health care professionals in offering personalized and meaningful care for patients with spinal disorders. The following review questions were answered: (1) Which CDSS tools can be identified in the current literature on spinal disorders? (2) What are the different purposes that the CDSS tools serve for spinal disorders? (3) How are these CDSS tools developed and assessed for effectiveness? and (4) What are the user’s perceptions and experiences regarding the use of CDSS tools?

## Methods

### Overview

This review was conducted using the Joanna Briggs Institute (JBI) methodological guidance for scoping review and reported according to the PRISMA-ScR (Preferred Reporting Items for Systematic Reviews and Meta-Analyses Extension for Scoping Reviews) statement [27,28]. The protocol for this review was registered in the Open Science Framework.

### Eligibility Criteria

The following inclusion criteria were used to determine study inclusion: (1) the study examined the CDSS use in patients with spinal disorders affecting the spinal column, cord, nerves, discs, or vertebrae in the cervical, thoracic, lumbar, or sacral regions of the spine and those with back pain, neuropathic pain, numbness, abnormal sensation, or tension caused by spinal issues; (2) all types of CDSS were considered, including integrated or independent systems, with purposes including diagnosis, disease or treatment prognosis, and treatment management of spinal disorders; (3) all participants were considered, with no restrictions placed on their cultural or racial background, geographic location, sex, or clinical management setting (acute or community); and (4) there were no restrictions placed on the study type, design, or source. The studies were excluded if they did not involve human participants, did not use a digitalized solution for ease of accessibility and use, were not applied in a clinical setting, or were reviews.

### Search Strategy

Both published and unpublished studies were located through PubMed, Embase, Cochrane, CINAHL, Web of Science, Scopus, ProQuest, and PsycINFO databases from inception until October 11, 2022. A limited initial search of PubMed was conducted to identify related articles and gather relevant keywords to develop a complete search strategy. The search strategy (Multimedia Appendix 1) was formed using the main concepts, including *clinical decision support system* and *spinal disorders*, combined with Boolean operators of *AND* and *OR*. The keywords and index terms were adapted for each database, and the reference lists of the included sources were screened for additional relevant studies. No limitations were placed on the sources’ language or date of publication to ensure that all relevant information on the topic was captured. In addition, sources of unpublished studies or gray literature, such as ClinicalTrials.gov, the International Standard Randomized Controlled Trial Number Register, the World Health Organization International Clinical Trials Registry Platform, and the Directory of Open Access Journals, were also searched.

### Source of Evidence Selection

Potential records were collated and uploaded to EndNote 20 (Clarivate), with duplicates removed [29]. Two independent reviewers (ZAT and QYCH) screened the titles and abstracts based on the eligibility criteria. The full text of potentially relevant studies was retrieved and further assessed for eligibility by both reviewers. The studies that did not meet the inclusion criteria were recorded and reported in the scoping review. Any disagreements between the 2 reviewers at each stage of the selection process were resolved through discussion or involving an additional reviewer (BB).

### Data Extraction and Synthesis

Data were extracted from the studies by 2 independent reviewers (ZAT and QYCH) using a data charting form adapted from the standardized data extraction tool of the JBI [27]. The extracted data included details about the participants, concept, context, study methods, and key findings relevant to the review questions. Iterative updates to the charting table allowed for the addition of valid unforeseen data [27]. We organized the research according to the applications examined and summarized the characteristics of each group, including the settings, participants, study designs, performance measures, and overall conclusions.

## Results

### Study Selection

**Figure 1:**
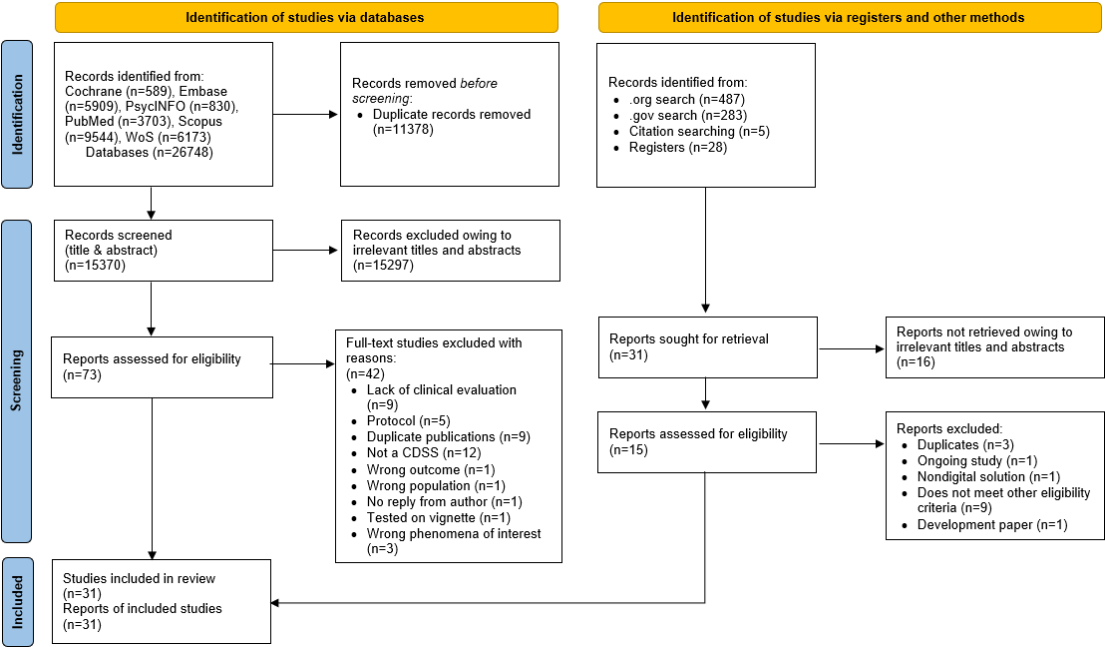
PRISMA (Preferred Reporting Items for Systematic Reviews and Meta-Analyses) flow diagram of study selection. CDSS: clinical decision support system.

A total of 26,828 records were identified from PubMed, Embase, Cochrane, CINAHL, Web of Science, Scopus, ProQuest, and PsycINFO databases. Of these, 73 (0.27%) full-text papers were retrieved after screening titles and abstracts and assessed against predetermined eligibility criteria (Figure 1); eventually, 31 (0.16%) studies were included for synthesis in this review, as summarized in Table 1. The studies were conducted in the United States (13/31, 42%), Australia (4/31, 13%), the Netherlands (3/31, 10%), Switzerland (2/31, 7%), Germany (3/31, 10%), Canada (1/31, 3%), Russia (1/31, 3%), Sweden (1/31, 3%), Ireland (1/31, 3%), South Korea (1/31, 3%), and the United Kingdom (1/31, 3%).

**Table 1:** A summary of the included CDSSs^a^ presented according to their purpose and application.

CDSS name and study		Country and setting		Study design (date)		Population	Sample size, N		Female, n (%)		Age (y), mean (SD)
**Preventing unnecessary imaging**											
	Choosing Wisely recommendation (Stanson Health), Chen et al [30], 2020	United States and single institution ambulatory clinic	CDSS testing: pre-post study (March 1, 2015, to April 30, 2017)		Patients with acute low back pain		Not reported	Not reported		Range 18 to 69	
	NEXUS^b^ clinical decision rule (Medweb), Goergen et al [31], 2006	Australia and single institution emergency department	CDSS testing: prospective cohort study (October 2001 to September 2002) with historical controls (June 2000 to July 2001)		Patients with cervical spine trauma		Cohort: 353 and control: 403	Cohort: 156 (45) and control: 190 (47)		Cohort: 32 (23-45)^c^ and control: 32 (24-49)^c^	
	Combined NEXUS criteria and CCSR^d^ CDSS, Hynes et al [32], 2020	Ireland, and single institution emergency department	CDSS testing: prospective cohort study (March to April 2017) with historical controls (March to April 2016)		Patients with cervical spine trauma		Not reported	Not reported		Not reported	
	ACP^e^ APS^f^ guideline derived CDSS, Ip et al [33], 2014	United States, and primary care service in an integrated health system with a quaternary care hospital and outpatient network	CDSS testing: prospective cohort study (2007 to 2010) with control cohort derived from NAMCS^g^		Patients with low back pain		Cohort: 21,445 and control: 2240	Cohort: 14,950 (69.7) and control: 1283 (57.3)		Cohort: 53.0 (15.6) and control: 50.5 (15.8)	
	ACR^h^ select tool (National Decision Support Company), Mallavarapu and Christiason [34], 2020	United States, and emergency department of a 204-bed community hospital	CDSS testing: interrupted time series from 12 months before and 10 months after modification		Patients with low back pain		Not reported	Not reported		Not reported	
	Choosing Wisely Canada CDSS, Min et al [35], 2017	Canada and single institution emergency department	CDSS testing: retrospective pre-post study from January 1, 2013, to May 31, 2016		Patients with acute low back pain		Not reported	Not reported		Not reported	
	ACR appropriateness criteria CDSS (Institute of Clinical Systems Improvement), Solberg et al [36], 2010	United States and multispecialty medical group primary care clinics	CDSS testing: retrospective pre-post study (2006 to 2007)		Patients requiring MRI^i^ spine		Cohort: 148 cases and control: 151 cases	Overall: 62%		57.8	
	Zafar et al [37], 2019	United States and tertiary academic health system with 8 PCP^j^ practices	CDSS testing: RCT^k^ with varying intervention periods; baseline period (March 1, 2012, to October 4, 2012), intervention period 1 (February 6, 2013, to December 31, 2013), and intervention period 2 (January 14, 2014, to June 20, 2014, and September 4, 2014, to January 21, 2015)		Physicians ordering imaging for patients with low back pain		108 PCPs	Not reported		Not reported	
**Diagnostic tool**											
	Benditz et al [38], 2019	Germany and single hospital orthopedic department	CDSS testing: cross-sectional correlational study		Patients with back pain		111	53 (47.7)		59.47 (15.81)	
	Benditz et al [39], 2021	Germany and single hospital orthopedic department	CDSS testing: cross-sectional study		Patients with back pain		86	40 (47)		51 (17)	
	Lin et al [40], 2006	United States and Europe: nationwide pain clinic in United States and clinics in Europe	CDSS development and testing: cross-sectional study		Patients with low back pain		180	Not reported		Not reported	
	Peiris et al [41], 2014	Australia and nationwide primary care clinics	CDSS development and testing: mixed methods study		Patients with back pain		Overall, not reported and 20 GPs^l^ (recruited for qualitative portion)	7 (35)		8 (40) patients aged <50 y, 8 (40) patients aged 50 to 59 y, and 3 (15) patients aged >60 y^m^	
	Kim et al [42], 2022	South Korea and single institution hospital	CDSS testing: cross-sectional study		Patients with postural spinal deformity		140	81 (57.86)		24.94 (17.36)	
	The Vertebral Compression Fracture tool, Wang et al [43], 2011	United States	CDSS development: cross-sectional study (not reported)		Patients with vertebral compression fractures		128	Not reported		Not reported	
**Prognostic tool**											
	The Seattle Spine Score (Virginia Mason Medical Center), Buchlak et al [44], 2017	United States and single high-volume hospital	CDSS development and testing: retrospective predictive modeling study		Patients with spinal deformity and those who had undergone surgery		136	100 (73.5)		63.2 (11.2)	
	Simple Brace Predictor (University of Alberta Edmonton), Chalmers et al [45], 2015	United States, and single institution scoliosis clinic	CDSS development and testing: retrospective chart review		Patients with adolescent idiopathic scoliosis		Training data set: 62 and test data set: 28	75 (83.3)		13.5 (1.7)	
	The Dialogue Support (Swedish Society of Spinal Surgeons), Fritzell et al [46], 2022	Sweden and a nationwide study (data from Swespine)	CDSS development and testing: retrospective chart review		Patients with lumbar disc herniation, lumbar spinal stenosis, degenerative disc disease, and cervical radiculopathy and those who underwent surgery.		87,494	Not reported		Not reported	
	Subgroups for Targeted Treatment (STarT) Back screening tool (Keele University), Hill et al [47], 2008	United Kingdom and 8 primary care general practices	CDSS development and testing		Patients with nonspecific back pain		CDSS development sample: 131 and validation sample: 500	CDSS development sample: 77 (60) and validation sample: 293 (59)		CDSS development sample: 44 (10.0) and validation sample: 45 (9.7)	
	SCOAP-CERTAIN^n^ tool (SCOAP-CERTAIN), Khor et al [48], 2018	United States and 15 Washington state hospitals	CDSS development and testing: prospective registry		Patients who have undergone lumbar spinal surgery		1583	944 (59.6)		61.3 (12.5)	
	SpineSage (University of Washington), Lee et al [49], 2014	United States, 2 academic institutions	CDSS development and testing retrospective chart review		Patients who had undergone spina surgery		1476	634 (43)		49.4 (20.0)	
	Cleveland Lumbar Spine Surgery risk calculator (Cleveland Clinic), Lubelski et al [50], 2021	United States and single tertiary care institution	CDSS development: retrospective chart review		Patients who had undergone lumbar spine surgery		2996	1386 (46)		58.3 (15.0)	
	Dartmouth Back Treatment Outcomes Calculator (Dartmouth College), Moulton et al [51], 2018	United States and multidisciplinary spine centers and web-based consumer reports subscribers	CDSS testing; cross-sectional study		Web-based subscribers of consumer reports and patients presenting with IDH^o^, SpS^p^, or DS^q^		1256 consumer participants and 68 patient participants	Consumer: 336 (27) and patient: 30 (44)		Consumer: 67 (9.3) and patient: 59 (16)	
	Schulthess Klinik Prognostic tool (Schulthess Klinik), Müller et al [52], 2021	Switzerland and single institution hospital	CDSS development: prospective cohort study		Patients with thoracic, lumbar, or cervical spinal degenerative disease		8374	4471 (53.4)		63.9 (14.3)	
	SCOAP-CERTAIN tool (SCOAP-CERTAIN), Quddusi et al [53], 2020	Netherlands and Dutch specialist short-stay spine center	External validation of prediction model		Patients with transforaminal lumbar interbody fusion or posterior lumbar interbody fusion		100	49 (49)		50.4 (11.4)	
	FUSE-ML (Machine Intelligence in Clinical Neuroscience & MICrosurgical Neuroanatomy laboratory), Staartjes et al [54], 2022	Multinational and multicenter (Switzerland, Netherlands, Italy, South Korea, France, and Austria)	CDSS development and testing		Patients who had undergone lumbar spinal fusion for degenerative disease		CDSS development sample: 817 and validation sample: 298	CDSS development sample: 468 (57.3) and validation sample: 192 (64.4)		CDSS development sample: 61.19 (12.3) and validation sample: 59.73 (12.6)	
**Treatment recommendation**											
	Benditz et al [38], 2019	Germany and single hospital orthopedic department	CDSS testing: cross-sectional correlational study		Patients with back pain		111	53 (47.7)		59.47 (15.81)	
	Byvaltsev and Kalinin [55], 2021	Russia and single hospital	CDSS testing: prospective cohort study with retrospective controls		Patients who had undergone lumbar spinal surgery		59 prospective cohort and 196 retrospective controls	Prospective cohort: 21 (35.6) and retrospective control: 59 (30.1)		Not reported	
	Downie et al [56], 2020	Australia and community pharmacy setting	CDSS development: mixed methods cross-sectional study		Patients with lower back pain		5 practicing community pharmacists	Not reported		Not reported	
	Back-UP (Horizon 2020), Jansen-Kosterink et al [57], 2021	Netherlands and community setting	CDSS testing: mixed methods study		Patients with chronic lower back pain		98 PCPs	47 (48)		48 (12.2)	
	Subaxial Injury Classification (SLIC) CDSS (Kubben), Kubben et al [58], 2011	Netherlands and not specified	CDSS development: Descriptive study		Patients with subaxial cervical spinal injury		Not reported	Not reported		Not reported	
	Peiris et al [41], 2014	Australia and nationwide primary care clinics	CDSS development and testing: mixed methods study		Patients with back pain		Overall, not reported; 20 GPs (recruited for qualitative portion)	7 (35)		8 (40) patients aged <50 y, 8 (40) patients aged 50 to 59 y, and 3 (15) patients aged >60 y	

^a^CDSS: clinical decision support system.

^b^NEXUS: National Emergency X-Radiography Utilization Study Group.

^c^Median (IQR).

^d^CCSR: Canadian Cervical Spine Rule.

^e^ACP: American College of Physicians.

^f^APS: American Pain Society.

^g^NAMCS: National Ambulatory Medical Care Survey.

^h^ACR: American College of Radiology.

^i^MRI: magnetic resonance imaging.

^j^PCP: primary care provider.

^k^RCT: randomized controlled trial.

^l^GP: general practitioner.

^m^One response was missing for age value.

^n^SCOAP-CERTAIN: Surgical Care and Outcomes Assessment Programme-Comparative Effectiveness Translational Network.

^o^IDH: intervertebral disc herniations.

^p^SpS: spinal stenosis.

^q^DS: degenerative spondylolisthesis.

### Study Characteristics

The use of CDSSs in spinal disorders is summarized into 4 major categories based on their primary purpose and application, as presented in Table 1: of 31 CDSSs, 8 (26%) were for the prevention of unnecessary imaging, 6 (19%) were for diagnostic applications, 11 (35%) were for prognostic applications, and 6 (19%) were for treatment recommendations. Only 5 (16%) of the 31 studies investigated user perceptions and experiences concerning the use of CDSSs [31,41,51,56,57].

### CDSSs for Preventing Unnecessary Imaging

Of the 31 CDSS studies reviewed, the implementation and results of the 8 (26%) CDSSs used to determine if radiologic imaging was necessary for patients with lower back pathologies [30,33-35,37], patients with cervical spine trauma [31,32], and patients in general [36] are presented in Table 2. The CDSSs were mainly embedded into the electronic health record system or the computerized physician order entry, apart from the guidelines proposed by Goergen et al [31], which used a physical report card and independent software. These CDSSs were often implemented in health care settings, such as the emergency departments, where patients with back pain or cervical spine trauma were first seen by the physicians. They functioned as alerts to remind physicians to consider whether spinal imaging is necessary and can take different forms, including hard-stop, soft-stop, and passive alerts. Hard-stop alerts aim to prevent the physician from proceeding with imaging orders that do not meet the guideline requirements. In contrast, soft-stop alerts may allow the physician to continue with the ordered imaging but require them to provide a reason. Passive alerts only require acknowledgment and do not require further user interactions. Although some studies did not specify the type of alert used, the information provided in the studies allowed for inference that all studies used a soft-stop alert, excluding 1 study that used a passive alert function [37].

**Table 2:** Type, features, and results of the CDSSs^a^ for preventing unnecessary imaging.

CDSS name and study	CDSS type	Features of the CDSS	Results
Choosing Wisely recommendation (Stanson Health), Chen et al [30], 2020	Knowledge based	Pop-up and soft-stop alert: Provides best practice advise when a CT^b^ scan, x-ray, or MRI^c^ of lumbar spine is ordered for a female patient aged 18 to 49 y or a male patient aged 18 to 69 y Alert suppressed for comorbidities; complicated back pain owing to trauma, cauda equina syndrome, spondylitis, disc herniation, history of back surgery; and disciplines, including neurosurgery, orthopedics, trauma surgery, anesthesiology, rheumatology, physical medicine and rehabilitation, oncology, and neurology	Post-CDSS implementation: Overall imaging rate decreased from 5.8% to 5.2% (9.6% decrease; *P*=.02) MRI imaging rate decreased from 1.8% to 1.5% (16.7% decrease; *P*<.01) No statistically significant differences in the rates of x-ray (*P*=.39) or CT (*P*=.88) orders Rationale for override A total of 64% used preset options: duration >6 weeks (37%), focal neurological deficit (14%), history of trauma (10%), previous spine surgery (1%), unexplained weight loss or insidious onset (1%), and unexplained fever or recent infection (1%) Free-text rationale (n=125, 36%); 56% were inappropriate
NEXUS^d^ clinical decision rule (Medweb), Goergen et al [31], 2006	Knowledge based	Guideline questionnaire based on NEXUS criteria and passive alert: Helps physicians to determine which patients to image and which imaging method (eg, plain radiography or helical CT) to use first	Compliance with CDSS and imaging guidelines: 40% (141/353) of patients were managed using the CDSS Of the 51 patients for whom the NEXUS guideline did not recommend imaging, 86% (43/51) did not receive any imaging Cervical spine imaging ordered: CDSS intervention group: 63.8% and control group: 78.5% (*P*=.01) Cervical spine imaging ordered (non-CDSS intervention): Non-CDSS intervention group^e^: 72.6% and control group: 78.5% (*P*=.11)
Combined NEXUS criteria and CCSR^f^ CDSS, Hynes et al [32], 2020	Knowledge based	Guideline questionnaire based on NEXUS and CCSR criteria and soft-stop alert Integrated in electronic imaging ordering system Helps physicians follow evidence-based guidelines when ordering cervical spine radiographs for patients who have experienced trauma Physicians asked to check boxes indicating which criteria the patient meets when ordering imaging	Cervical spine radiograph orders: Preintervention: 182 Postintervention: 126 (*P*<.001) Proportion of requests meeting NEXUS or CCSR criteria: Preintervention: 76.7% Postintervention: 99.2% (*P*<.001)
ACP^g^ APS^h^ guideline derived CDSS, Ip et al [33], 2014	Knowledge based	Guideline questionnaire based on ACP or APS criteria and soft-stop alert Integrated into the CPOE^i^ system Provides real-time decision support to physicians for imaging orders based on the patient’s clinical history	Lumbar spine MRI orders: Preintervention: 5.3% (443/8437) Postintervention: 3.7% (477/13,008; *P*<.001) Outpatient MRI orders 30 d after: Preintervention: 2.2% Postintervention: 2.7% (*P*=.03) LBP^j^-related visits that resulted in an MRI within 30 d of the index visit, accounting for imaging that was ordered by specialists Preintervention: 8.9% Postintervention: 7.8% (*P*=.002)
ACR^k^ select tool (National Decision Support Company), Mallavarapu and Christiason [34], 2020	Knowledge based	Guideline questionnaire based on ACR criteria and soft-stop alert Integrated into the electronic medical health system The *free text* field, which allowed providers to bypass the ACR select tool within EHRs^l^, has been removed to increase provider adherence to the tool	Preintervention: 13 scans/mo and postintervention: 11.6 scans/mo (*P*=.54)
Choosing wisely Canada CDSS, Min et al [35], 2017	Knowledge based	Guideline questionnaire based on recommendations from the Canadian Association of Emergency Physicians, the College of Family Physicians of Canada, Occupational Medicine Specialists of Canada, the Canadian Association of Radiologists, and the Canadian Spine Society, and soft-stop alert Integrated with the CPOE Physicians must select a suspected diagnosis when ordering an imaging test for LBP If a physician selects *other* as the suspected diagnosis, they will need to provide an explanation for ordering the imaging test outside the established appropriateness criteria	Proportion of LBP patients with imaging order fell significantly compared with preimplementation baseline after CDSS implementation Median: 22% decreased to 17% Mean: 23% decreased to 18%; (*P*<.001) Imaging ordering patterns A total of 60% (26/43) of the physicians reduced their ordering of imaging tests. Imaging orders placed 1 to 30 d after LBP presentation Preintervention: 2.3% and postintervention: 2.2% (*P*=.97) ED^m^ revisit: preintervention 8.2% and postintervention 6.9% (*P*=.17)
ACR appropriateness criteria CDSS (Institute of Clinical Systems Improvement), Solberg et al [36], 2010	Knowledge based	Guideline questionnaire based on ACR criteria and soft-stop alert Integrated within the EHR system and requires physicians to enter a reason for every order placed. No safeguards were in place to prevent orders from being placed even if they did not meet certain criteria. Physicians received little feedback on the outcomes of their orders.	Volume of spine MRI ordered decreased by 20% Impact of CDSS on patient’s health after spine MRI increased from 14% to 30% (*P*=.18)
Zafar et al [37], 2019	Knowledge based	Guideline based on ACP and APS criteria and soft-stop or passive alert Embedded in CPOE, the CDS^n^ algorithm screen the lumbar spine MRI orders for adherence to the guideline Intervention groups: periodic CDSS report cards vs real-time CDSS alerts vs both	Likelihood of placing lumbar spine MRI orders at the time of LBP presentation when compared with baseline CDSS report cards: 38% lower likelihood Real-time CDSS alerts: not associated with any change (*P*=.59)

^a^CDSS: clinical decision support system.

^b^CT: computed tomography.

^c^MRI: magnetic resonance imaging.

^d^NEXUS: National Emergency X-Radiography Utilization Study.

^e^Imaging guidelines given in a form of pocket card and posters, with small group teaching sessions.

^f^CCSR: Canadian Cervical Spine Rule.

^g^ACP: American College of Physicians.

^h^APS: American Pain Society.

^i^CPOE: Computerized provider order entry.

^j^LBP: low back pain.

^k^ACR: American College of Radiology.

^l^EHR: electronic health record.

^m^ED: emergency department.

^n^CDS: clinical decision support.

The included studies reported ≥1 of the following outcomes: change in the frequency of imaging order, change in the frequency of imaging order 1 to 30 days after LBP presentation, and adherence to order guidelines. All studies reported a decrease in imaging ordered on the initial presentation of LBP after the implementation of a CDSS, although the decrease was not clinically relevant in some studies [30,34]. Ip et al [33] reported a notable increase (22.7%; *P*=.03) from 2.2% (188/8437) to 2.7% (352/13,008) in the lumbar spine–magnetic resonance imaging (LS-MRI) ordered by outpatient specialists within 30 days of the patient’s primary care visit. This increase may be explained by the fact that the CDSS intervention was implemented in the primary care setting but not in the outpatient setting. However, when considering the total percentage of the LS-MRI orders for LBP visits before and after CDSS implementation, there was a statistically significant decline (12%; *P*=.002) from 8.9% (753/8437) to 7.8% (1009/13,008) in imaging orders after adjusting for outpatient specialist orders.

Zafar et al [37] compared the outcomes of different CDSS deliveries for LS-MRI orders [37]. The CDSS report cards that were generated every 4 to 6 months led to fewer magnetic resonance imaging (MRI) orders (50/1739, 2.9%) for cases compared with immediate CDSS alerts (94/2021, 4.7%).

Furthermore, CDSSs, generally, were reported to improve adherence to imaging guidelines. For example, Hynes et al [32] reported a 99.2% adherence rate to the established imaging guidelines after CDSS implementation (125 indicated imaging out of 126 total imaging), an increase of 22.5% (76.7 to 99.2%) from preimplementation [32]. Similarly, Solberg et al [36] discovered a reduction of 20% in the volume of MRI spine orders and an increase in the appropriateness of MRI spine orders based on health impacts [36].

### Diagnostic CDSS

Of the 31 studies reviewed, 6 (19%) explored diagnostic CDSSs (Table 3) and 3 (10%) examined the accuracy of CDSS compared with expert or *gold standard* diagnoses [38,39,42]. A moderate agreement was found between the CDSS and expert diagnoses for back pain (Cramer V=0.424) [38]. A higher agreement of 67% (58/86) of the cases between the CDSS and expert diagnosis (Cramer V=0.711) was found for patients with spinal disorders in general [39]. Another study by Lin et al [40] found that a CDSS performed a diagnosis comparable to that of experts and correctly recommended 75.82% of diagnoses based on gold-standard criteria [40]. In a recent study by Kim et al [42], the CDSS diagnosis demonstrated a 94% agreement with the gold-standard radiographic assessment for scoliosis, with higher agreement reported for patients within the normal and mild postural deformation range [42].

**Table 3:** Type, features, and results of the CDSSs^a^ for diagnostic support.

CDSS name and study	CDSS type	Features of the CDSS	Results
Benditz et al [38], 2019	Knowledge based	Questionnaire-based CDSS A computerized tool with disease-specific algorithms cascading the next best questions leading to the most probable diagnosis and actions	Diagnosis of CDSS compared with spinal surgeons: Cramer V=0.424^b^; *P*<.001
Benditz et al [39], 2021	Knowledge based	Decision tree algorithm and app-based questionnaire Questionnaire will ask the patient to identify the location of their pain and present dichotomous questions to suggest a diagnosis. If the patient scores >65% on these questions, the diagnosis is confirmed. If not, the questionnaire will ask additional questions about the second most likely diagnosis. If the patient still scores <65% after these questions, they are advised to consult with a physician.	Diagnosis of CDSS compared with spinal surgeons: Cramer V=0.711^b^; *P*<.001 Concordance: 67.4% A total of 15.1% overestimated A total of 7% underestimated
Lin et al [40], 2006	Knowledge based	Knowledge from 2 highly experienced physical therapists and web-based questionnaire Patients can start a self-diagnosis session with or without clinician’s assistance. Questions regarding specific pain symptom or assessment will be presented through typically 13 to 15 web pages, depending on the number of follow-up questions triggered. After completion of the questions, a diagnosis that may consist of ≥1 parts, based on patient information, clinical evidence provided by the user, and system’s rule activation, will be generated and the clinician can override any parts of the diagnosis. The explanatory panel can be activated by the clinician to review the system’s reasoning process. The clinician can add, remove, or modify an existing rule in reference to its observation, decision outcome, or certainty level.	Not reported
Peiris et al [41], 2014	Knowledge based	Recommendations from 15 guidelines for back pain management After excluding serious pathology, the CDSS will continue to assess for the most probable diagnosis and treatment through a series of questions. A personalized information sheet will be printed.	Not reported
Kim et al [42], 2022	Nonknowledge based	Computer vision–based posture analysis system The CDSS uses a Kinect sensor and specialized software to analyze a person’s skeletal structure and gait. The CDSS captures an image and records a moving video of the participant. The software then identifies the participant’s joints and uses them to determine the skeletal structure and gait. Furthermore, it uses a set of algorithms to judge the probability of scoliosis by analyzing the curvature of the participant’s central coronal axis, which is determined by a line connecting the eyes, shoulders, and pelvis. The CDSS classifies scoliosis as normal (≤3 mm curvature), 20% scoliosis (3 mm to 10 mm curvature), or 50% scoliosis (>10 mm curvature).	Postural deformations: assessed with 94% accuracy (comparable with radiographic assessments) Normal or mild scoliosis: conformity assessment accuracy of 98.57% CDSS’s diagnostic accuracy for scoliosis was 0.94, with the most influential factors being spinal curvature and pelvis height, which accounted for 79.97% and 19.86% of the variance in the data, respectively
Vertebral Compression Fracture tool, Wang et al [43], 2011	Knowledge based	Logistic regression and web-based checklist Uses checklists for dichotomous and nondichotomous discrete variables based on MRI^c^ features to generate a probability of malignancy and a text report. The model captures inputs from these variables to make its assessment.	Not reported

^a^CDSS: clinical decision support system.

^b^Interpretation of Cramer V effect size measurement of association: effect size ≤0.2: weak association, <0.2 effect size ≤6: moderate association, and effect size >0.6: strong association.

^c^MRI: magnetic resonance imaging.

### Prognostic CDSS

Of the 31 CDSS studies reviewed, 11 (35%) prognostic CDSS studies (Table 4) were knowledge based [44,45,47-54,59], with regression-based predictive algorithms. White-box models were used across all studies; most CDSSs were presented as web-based calculators, whereas others were presented as independent software. Prognostic CDSSs are used for various purposes, most commonly to predict the likelihood of complications, functional outcomes, pain, and quality of life following spinal surgery (8/11, 73%). Other purposes included predicting the outcome of brace treatment for adolescent idiopathic scoliosis (1/11, 9%), the risk of back pain chronicity (1/11, 9%), and treatment outcomes between surgical and nonsurgical options for spinal disorders (1/11, 9%). Regarding rigor, external validation was only available for 3 (27%) CDSS models (FUSE-ML, Surgical Care and Outcomes Assessment Programme-Comparative Effectiveness Translational Network Tool, and STarTBack), and an impact study was only performed for the StarTBack model.

A total of 2 key aspects, namely discrimination and calibration, are often measured to evaluate the performance of a model. Discrimination can be assessed using various measures such as area under the receiver operating characteristics, accuracy, sensitivity, positive predictive values, negative predictive values, *R*^2^ measure or value, or any specific statistic measure, such as Nagelkerke, c-index, mean absolute error, and root mean square error. In contrast, calibration can be evaluated using techniques such as calibration plot, calibration intercept and slope, and the Hosmer-Lemeshow chi-square statistic.

The impact study was the only study that conducted a clinical impact testing follow-up, as reported by Foster et al [59]. This study developed an innovative web-based calculator that assesses patients’ risk of developing chronic LBP and offers tailored treatment options for each risk stratum. Results from the impact study revealed small but significant improvements (*P*=.03) in Roland-Morris disability scores, with a mean difference of 0.71 (95% CI 0.06-1.36) compared with usual care after 6 months of implementation. Furthermore, the group with a higher risk of developing chronic LBP experienced a large and clinically significant improvement. Work absence was also reduced by 50% (4 days instead of 8 days; *P*=.03), and there was a 30% decrease in prescriptions for sickness certificates (45/368, 12.2% vs 40/554, 7.2% cases; *P*=.03).

**Table 4:** The predicted outcome, input variables, and internal and external validation of prognostic CDSSs^a^.

CDSS name and study	Outcome	Input variables	Internal validation		External validation	
			Discrimination	Calibration	Discrimination	Calibration
Seattle Spine Score (Virginia Mason Medical Center), Buchlak et al [44], 2017	Percentage of likelihood of complications occurring within 30 d	Age, BMI, gender, smoking status, anemia, diabetes, and hypertension	AUC^b^: 0.71 Accuracy: 75%	HLT^c^: 3.692; *P*=.88	Not reported	Not reported
Simple brace predictor (University of Alberta Edmonton), Chalmers et al [45], 2015	Scoliosis progression	In-brace correction and scoliometer measurements	Accuracy: 75%	Not reported	Not reported	Not reported
The Dialogue support (Swedish Society of Spinal Surgeons), Fritzell et al [46], 2022	GA^d^ pain and satisfaction	Diagnosis group, operated levels, clinical department type, age, gender, employment, disability or retirement pension, health profile, smoking history, previous spinal surgery, quality of life, comorbidity, back-specific information, walking distance, duration and severity of preoperative pain in legs and back, and ODI^e^	Pain AUROC^f^: 0.67-0.68 Satisfaction AUROC: 0.60-0.67	Calibration plots: high degree of concordance	Not reported	Not reported
Subgroups for Targeted Treatment (STarT) Back screening tool (Keele University), Hill et al [47], 2008	Risk of chronicity	Referred leg pain, comorbid pain, disability, bothersomeness, catastrophizing, fear, anxiety, and depression	AUROC: 0.74-0.92	Not reported	Sensitivity: 80.1 Specificity: 65.4 Positive likelihood ratios: 2.32 Negative likelihood ratios: 0.30	Not reported
SCOAP-CERTAIN^g^ tool (SCOAP-CERTAIN), Khor et al [48], 2018, external validation, Quddusi et al [53], 2020	Functional outcome, back pain, and leg pain	Age, gender, insurance, race or ethnicity, ASA^h^ score, smoking status, prior surgery, spondylolisthesis, disc herniation, postlaminectomy, failed back syndrome, stenosis, pseudarthrosis, radiculopathy, prescription opiate use, asthma, baseline ODI and NRS^i^ score	AUROC ODI: 0.66 Back pain: 0.79 Leg pain: 0.69	Calibration plots	ODI AUROC: 0.71 Sensitivity: 0.64 Specificity: 0.65 Accuracy: 0.65 PPV^j^: 0.84 NPV^k^: 0.4 F1-score: 0.49 NRS back pain AUROC: 0.72 Sensitivity: 0.81 Specificity: 0.48 Accuracy: 0.73 PPV: 0.84 NPV: 0.42 F1-score: 0.44 NRS-Leg pain AUROC: 0.83 Sensitivity: 1.00 Specificity: 0.38 Accuracy: 0.85 PPV: 0.84 NPV: 1.00 F1-score: 0.54	ODI calibration intercept: 1.08 Calibration slope: 0.95 HLT: *P*=.002 Brier score: 0.22 NRS back pain Calibration intercept: 1.02 Calibration slope: 0.74 Brier score: 0.19 NRS-Leg pain Calibration intercept: 1.08 Calibration slope: 0.95 Brier score: 0.12
SpineSage (University of Washington), Lee et al [49], 2014	Occurrence of medical complications after spinal surgery	Age, gender, smoking status, alcohol use, diabetes, BMI, insurance status, surgical approach, revision surgery, region, diagnosis, surgical invasiveness, and medical comorbidity	Any medical complications: AUROC: 0.76 Any major medical complications: AUROC: 0.81	Not reported	Not reported	Not reported
Cleveland Lumbar Spine Surgery risk calculator (Cleveland Clinic), Lubelski et al [50], 2021	Postoperative ED^l^ visit or readmission, and quality of life	Race, marital status, symptom duration, BMI, CCI^m^, foraminal stenosis, disc herniation, spondylolisthesis, radiculopathy, procedures (eg, ALIF^n^, PLIF^o^, and TLIF^p^, posterolateral lumbar fusion, and decompression), number of operated levels, preoperative Pain and Disability Questionnaire score, and EQ-5D^q^	30-d visits to the ED c-index^r^: 0.63 30-d readmission c-index: 0.66 90-d reoperation related to infection c-index: 0.73 1-y postoperative EQ-5D outcome c-index: 0.84	Calibration plot	Not reported	Not reported
Dartmouth back treatment outcomes calculator (Dartmouth College), Moulton et al [51], 2018	Physical function, pain, sleep, sex life, and satisfaction with symptoms	Age, gender, height, weight, bothersomeness, back and leg pain, numbness, leg weakness, leg pain while sitting, activity level, employment status, smoking status, duration of sciatica worsening, work compensation, hypertension, depression, education level, physical therapy, sleep, and sex life	Not reported	Not reported	Not reported	Not reported
Schulthess Klinik Prognostic Tool (Schulthess Klinik), Müller et al [52], 2022	Back and leg pain, COMI^s^, impairment, symptom-specific well-being, quality of life, social disability, and work disability	Preoperative axial and peripheral pain, catastrophizing, fear-avoidance beliefs, comorbidity, age, BMI, nationality, previous spinal surgery, type and spinal level of intervention, number of affected levels, and surgeon seniority	Not reported	Not reported	Not reported	Not reported
FUSE-ML (Machine Intelligence in Clinical Neuroscience & MICrosurgical Neuroanatomy laboratory), Staartjes et al [54], 2022	Functional outcome and back and leg pain	Age, gender, surgical indication, index level, height, weight, BMI, smoking status, ASA score, preoperative opioid use, bronchial asthma, prior thoracolumbar spinal surgery, race or ethnicity, surgical approach, pedicle screw insertion, ODI or COMI, and leg and back NRS	ODI or COMI AUROC: 0.75 Accuracy: 0.70 Sensitivity: 0.70 Specificity: 0.70 PPV: 0.88 NPV: 0.43 Back pain AUROC: 0.71 Accuracy: 0.68 Sensitivity: 0.68 Specificity: 0.63 PPV: 0.91 NPV: 0.26 Leg pain AUROC: 0.72 Accuracy: 0.74 Sensitivity: 0.77 Specificity: 0.58 PPV: 0.90 NPV: 0.34	ODI or COMI Calibration intercept: 0.00 Calibration slope: 0.89 Back pain Calibration intercept: 0.00 Calibration slope: 0.86 Leg pain Calibration intercept: 0.00 Calibration slope: 0.84	ODI or COMI AUROC: 0.67 Accuracy: 0.61 Sensitivity: 0.59 Specificity: 0.66 PPV: 0.81 NPV: 0.39 Back pain AUROC: 0.72 Accuracy: 0.70 Sensitivity: 0.72 Specificity: 0.64 PPV: 0.90 NPV: 0.34 Leg pain AUROC: 0.64 Accuracy: 0.71 Sensitivity: 0.76 Specificity: 0.42 PPV: 0.88 NPV: 0.23	ODI or COMI Calibration intercept: −0.07 Calibration slope: 0.63 Back pain Calibration intercept: −0.38 Calibration slope: 1.10 Leg pain Calibration intercept: 0.14 Calibration slope: 0.49

^a^CDSS: clinical decision support system.

^b^AUC: area under the curve.

^c^HLT: Hosmer-Lemeshow Test.

^d^GA: general anesthesia.

^e^ODI: Oswestry Disability Index.

^f^AUROC: area under the receiver operating characteristics.

^g^SCOAP-CERTAIN: Surgical Care and Outcomes Assessment Programme-Comparative Effectiveness Translational Network.

^h^ASA: American Society of Anesthesiologists.

^i^NRS: Numeric Rating Scale.

^j^PPV: positive predictive value.

^k^NPV: negative predictive value.

^l^ED: emergency department.

^m^CCI: Charlson Comorbidity Index.

^n^ALIF: anterior lumbar interbody fusion.

^o^PLIF: posterior lumbar interbody fusion.

^p^TLIF: transforaminal lumbar interbody fusion.

^q^EQ-5D: EuroQOL-5D.

^r^C-index: concordance.

^s^COMI: Core Outcome Measures Index.

### CDSS for Treatment Recommendation

Of the 31 CDSS studies reviewed, studies exploring the use of CDSS for treatment recommendations for spinal disorders were divided into 2 categories based on their focus: 2 (6%) CDSSs for recommendations for spinal surgery [55,58] and 4 (13%) CDSSs for treatment of LBP [38,41,56,57] (Table 5). All CDSSs were knowledge based, except for 1, which was structured on medical ontology and fuzzy logic principles [55]. The system inputs required to generate personalized treatment recommendations include symptoms, clinical findings, and instrumental findings.

Byvaltsev and Kalinin [55] studied using a CDSS to recommend total disc replacement, minimally invasive rigid stabilization, and open rigid stabilization [55]. The researchers observed lower pain levels and improved functional status 6 months after surgery among those who received treatment recommendations using the CDSS [55]. Those who underwent minimally invasive rigid stabilization had better outcomes 3 months after surgery [55]. In the work of Benditz et al [38], although 49.6% (55/111 cases) of the treatment recommendations made by the CDSS were consistent with those of spinal surgeons, 36% (40/111) were overestimated and 14.4% (16/111) were underestimated [38]. In contrast, a study by Downie et al [56] revealed that CDSS recommendations were highly concordant with those made by pharmacists for cases involving self-care (18/20, 90%), medications (25/25, 100%), and referral advice (22/25, 88% [56]).

**Table 5:** Type, features, and outcomes measured for the CDSS^a^ for treatment recommendation.

Study and CDSS name	CDSS type	Features of the CDSS	Outcomes measured	Results
Benditz et al [38], 2019	Knowledge based	Questionnaire-based CDSS A computerized tool with disease-specific algorithms cascading the next best questions leading to the most probable diagnosis and actions	Association between the diagnoses and treatment recommendation of the tool and the physician’s diagnosis	Significant correlation with small-to-medium effect between the DSS^b^ and the medical recommendation. Cramer V=0.293; *P*=.02 Concordance: 49.6% Overestimated: 36% Underestimated: 14.4%
Byvaltsev and Kalinin [55], 2021	Nonknowledge based	Semantic network structured based on the medical ontology and fuzzy logic principles Computer-assisted electronic checklist and recommendations, which uses preoperative instrumental data on lumbar segments of patients with degenerative diseases	Pain using visual analog scale Lower limbs Lumbar spine ODI^c^	For patients who underwent total disc replacement, pain syndrome level and functional status were comparable before surgery, on discharge and 3 mo after surgery (*P*>.05). A total of 6 mo after the surgery, there was a decrease in pain intensity in the lower limbs (*P*=.02) and lumbar spine (*P*=.03) and an increase in functional status by ODI (*P*=.02) in the CDSS group. In the CDSS patients group who underwent minimally invasive rigid stabilization, there was a decrease in pain intensity in the lower limbs (*P*=.01 for both 3 mo and 6 mo after surgery) and in the lumbar spine (*P*=.03 and *P*=.02 for 3 mo and 6 mo after surgery, respectively) and an increase in functional status by ODI (*P*=.01 and *P*=.03 for 3 mo and 6 mo after surgery, respectively). For patients who underwent open rigid stabilization, pain syndrome level and functional status were comparable (*P*>.05) before surgery, on discharge and 3 mo after the surgery. A total of 6 mo after surgery, there was a decrease in pain intensity in the lower limbs (*P*=.04) and lumbar spine (*P*=.03) and an increase in functional status by ODI (*P*=.01) in the group using CDSS.
Downie et al [56], 2020	Knowledge based	Decision tree app–based CDSS It consists of a knowledge base, reasoning engine, and interface. An advice report will be generated after the history and screening inputs. The pharmacist may add any key message or modify the advice.	Qualitative-based CDSS: Ease of use, consistency (of visual language or interaction model), system visibility, navigation or workflow, content, clarity, and acceptance System usability scale Level of acceptance of clinical reasoning and decision support	Ease of use: mostly negative sentiments (16/26, 62%) Consistency: mostly neutral sentiments (7/13, 54%) Visibility: mostly negative sentiments (7/16, 44%) Navigation or workflow: mostly neutral sentiments (12/16, 75%) Content: mostly positive sentiments (12/27, 44%) Clarity: mostly neutral sentiments (9/15, 60%) Acceptance: mostly positive sentiments (34/49, 69%) System usability scale Overall system usability: excellent (mean 0.92, SD 6.5), with acceptance rated as good to excellent. CDSS-pharmacists' agreement: Self-care recommendations: 90% (18/20) Medicines recommendations: 100% (25/25) Referral advice :88% (22/25) Pharmacists expressed uncertainty when screening for serious pathology in 40% (10/25) of the cases. Pharmacists requested more direction from the CDSS in relation to automated prompts for user input and page navigation.
Back-UP (Horizon 2020), Jansen-Kosterink et al [57], 2021	Knowledge based	Binary logistic regression Short questionnaires were completed by patients that stratified them into 1 of the 3 outcome groups. Targeted interventions were recommended for each outcome group.	Qualitative-based CDSS: Factors that promote or hinder the acceptance of clinicians toward CDSS use	Reason to use a complex CDSS: Improve care of patients (assessment, n=20) Curiosity to test and use the CDSS, to see for themselves what the value of the system is (n=19) Expectation of increase in efficiency because of reduction of workload and time and allowing them to reorganize work (n=18) Support during decision-making (n=17) Patient empowerment (n=14) Work consistently with evidence-based medicine (n=8) Perceived the technology as friendly to use (n=3) Barriers to using a complex CDSS: Worried about their own clinical practice and autonomy; physicians are reluctant to use a CDSS when it interferes too much with clinical practice (n=18) Do not want to use a CDSS when it comes at an increase in time and costs (n=18) The fear that the CDSS does not work correctly (n=17) A too generic approach (n=15) A lack of effectiveness and added value (n=11) Hampering personal contact with the patient (n=8) Data and security issues (n=8) Capitalizing on health care (n=4) Lack of trust (n=3) If the use of CDSS is imposed by external parties, such as health care insurance companies (n=3)
SLIC^d^ CDSS (Kubben), Kubben et al [58], 2011	Knowledge based	Decision tree app–based CDSS Offers evidence-based algorithms (eg, burst fractures, central cord syndrome, facet fracture dislocation, facet subluxation, and hyperextension injury) based on the Subaxial Injury Classification system	Not reported	Not reported
Peiris et al [41], 2014	Knowledge based	Recommendations from 15 guidelines for back pain management After excluding serious pathology, the CDSS will continue to assess for the most probable diagnosis and treatment through a series of questions. A personalized information sheet will be printed.	Frequency of use of the web-based tool by physicians Acceptability by physicians	Acceptability by physicians Considered that back pain is easy to manage and the use of CDSS could insult their skills Found CDSS useful for patient reassurance and minimizing complex tests or treatment. Suggestions for improvement: Increase comprehensiveness of advice for complex pain management and referral and allow customization of advice Integration of software systems and easy navigation

^a^CDSS: clinical decision support system.

^b^DSS: decision support system.

^c^ODI: Oswestry Disability Index.

^d^SLIC: Subaxial Injury Classification.

### User’s Perception and Experience

Of the 31 CDSS studies reviewed, 5 (16%) studies examined the user acceptability of CDSS use and gathered feedback for improvement [31,41,56,57]. User perceptions were mixed, with the most receptive toward CDSS use [41,56,57] because it provides evidence-based content to support patient care and empowerment by involving patients in decision-making. Some perceived the use of CDSS as additional work [31], while others doubted the tool’s accuracy owing to the complexity of LBP [41]. However, in cases where physicians felt that complex treatment or imaging was not recommended, CDSSs were found helpful in supporting their recommendations and reassuring patients about the decision [41]. Furthermore, the physicians were more likely to use CDSS if it lightened their workload or improved their efficiency [57].

## Discussion

### Principal Findings

We identified 4 major applications of the CDSS: preventing unnecessary imaging, aiding diagnosis, aiding prognosis, and suggesting treatment options. Only 2 studies used non–knowledge-based algorithms for diagnosis and treatment recommendations, whereas knowledge-based algorithms were the most commonly applied approach. Common input variables included age, gender, height, smoking status, education level, employment status, race or ethnicity, medical comorbidities, preoperative pain and disability, previous spinal surgery, symptom duration, surgical approach and intervention, BMI, American Society of Anesthesiologists score, and surgical diagnosis.

### CDSS for Preventing Unnecessary Imaging

MRI detects soft tissue abnormalities [60], but the increased cost, time, and logistical demands compared with other imaging techniques make its use inconsistent with value-based care for nonspecific indications [61]. The National Emergency X-Radiography Utilization Study criteria, Canadian Cervical Spine Rule, and American College of Physicians and American Pain Society guidelines were created to direct the diagnosis and treatment of back pain and suspected spinal injury [62,63]. However, adherence to these guidelines is poor owing to *defensive medicine*, the continued use of unnecessary imaging to avoid missing serious pathologies [35,64].

Integrated CDSSs offer 2 benefits. First, they act as gatekeepers by adding an extra step before imaging is ordered [35]. Second, they educate or remind physicians of the existing guidelines, reducing the need to memorize multiple protocols [35]. Most studies have found that using the CDSS decreases the number of imaging tests ordered both at the time of the initial LBP visit and up to 30 days after the visit. However, other studies have not found a significant decrease in imaging orders, suggesting a potential mistrust of the system or a lack of awareness of imaging guidelines [34]. Furthermore, an insignificant decrease in imaging order may arise from the decision to use computerized tomography or x-ray instead of MRI, which could be more appropriate for some patients [30].

The use of alert-based CDSS raised concerns about alert fatigue, where repeated alerts may lead to physicians ignoring system prompts. Unnecessary imaging frequency was reduced when CDSS-generated report cards were distributed to physicians every 4 to 6 months compared with real-time alerts [37]. Furthermore, the ease of use of CDSS can hinder proper imaging if separate software is required, requiring the physician to toggle between the ordering and the CDSS system. In addition, the lack of real-time consequences for ignoring prompts may contribute to the continuation of unnecessary imaging practices.

### Diagnostic CDSS

In general, diagnostic CDSSs operate through questionnaires that generate probable diagnoses. CDSS-generated diagnoses were found to be primarily concordant with expert or gold-standard recommendations, indicating potential feasible use. Despite its ability to provide reliable diagnoses, most studies still recommend using the diagnostic CDSS as an aid instead of a replacement for the expertise and judgment of trained and experienced health care professionals [38,39,43]. In addition, patient-provider interactions are essential, and a human connection is a part of building a healing and therapeutic relationship [65]. Health care providers can assess a patient’s physical and emotional well-being better than a machine, which is only as good as its algorithm. As an aid, diagnostic CDSS could allow a brief initial assessment of the patient’s condition and assist in triaging, allowing patients with critical spinal disorders to receive early attention [38,39].

To ensure generalizability and continued validity of the CDSS, it is crucial that regular updates with the latest evidence-based information be made available to the system [40]. Meanwhile, given the lack of non–knowledge-based CDSS for spinal diagnostic purposes, AI or machine learning algorithms should be explored. The potential of AI in the field of diagnosis remains to be fully tapped, especially in the areas of computer vision and image recognition. There are promising signs of the increased prominence of diagnostic CDSSs and their ability to produce faster and more accurate findings [66].

### Prognostic CDSS

All the included prognostic CDSS studies used white-box models. This model allows for the adaptation and modification of variables to identify areas for optimization to improve the outcomes [67]. Traditional statistical methods for prognostic modeling use simpler computation methods that allow insight into causal effects [67]. In contrast, machine learning methods are often referred to as black-box models owing to the computational complexity that allows for fast and accurate predictions but at the cost of transparency. Previous research has shown that machine learning models may perform poorer than traditional statistical methods, suggesting that this tradeoff is not justified [68]. The poorer performance may have resulted from using low-dimensional data; however, with the increasing availability of high-dimensional data and repositories of large data sets, such as biomarkers and imaging techniques, machine learning could have a competitive advantage over traditional statistics [54].

The prognostic CDSS systems are currently available as independent programs, as most are in the process of development or testing, and specialized sets of algorithms and flexibility for adjustments are required. Such an implementation could also be intentional to ease access for the users of a different electronic system, reduce the cost of integration, and ensure the confidentiality of data [10].

The prognostic CDSSs reviewed in our study were fragmented in their methodology, and none were ready for clinical implementation. The emergence of prognostic models employing AI and big data has been on the rise. However, reviews have identified poor standardization and quality of their development [69,70]. Previous reviews found that most prognostic model research ends with model development, with only a small number of studies performing external validation and even fewer conducting impact studies [70]. This aligns with the findings of our review, in which the included studies were found not to adhere well to standards, limiting the model’s validity, generalizability, and application in real-world clinical settings. Only 2 (6%) of the 31 included studies [52,54] used a reporting guideline, namely the *Transparent Reporting of a Multivariable Prediction Model for Individual Prognosis or Diagnosis* statement [71]. Future developments should adhere to the Prognosis Research Strategy prognostic model research framework, which emphasizes model development, external validation, impact testing [72], and reporting guidelines to ensure standardization and generalizability of the models.

The predictive ability of prognostic models is expected to weaken with time owing to changes in diagnostic and treatment approaches [72]. Therefore, it may be more beneficial to improve and recalibrate existing models instead of developing new models. In addition, including biomarkers and imagery data may improve model performance, but caution should be taken to address issues such as class imbalances, missing data, and the need for adequate validation [54]. Although adding more variables to a model can increase its predictive power, it can also make the model less user-friendly. To balance the tradeoff between accuracy and user-friendliness, parsimonious models that include only the most important or highly correlated predictors of the outcome are preferred. Techniques such as recursive feature elimination, principal component analysis, factor analysis, and multidimensional scaling can be used to identify key predictors [73].

### CDSS for Treatment Recommendation

According to Benditz et al [38], only 49.6% of the treatment recommendations made by the CDSS agreed with those of the physicians [38]. Although this low level of concordance may be seen as a problem and may affect confidence in the use of the CDSS, it is important to note that concordance is not necessarily the best indicator of performance; instead, testing the clinical effects of treatment options recommended by the CDSS may be a more accurate method to assess its performance.

Suggestions to improve the acceptance and usability of CDSSs include integrating them into the existing workflow and clinical decision-making processes [41]. This integration eases access to evidence-based information, encouraging use and adherence to the best practice guidelines [58].

Although the CDSS has been widely accepted for recommending treatment or management of spinal disorders, concerns and suggestions have been raised. The top barrier to CDSS use is interference with physician autonomy [57]. The physicians may feel threatened by CDSS recommendations and worry that they may eventually diminish their role in the care process [74], leading to questions about their competence [41]. In addition, ease of use is a common barrier; some physicians have negative sentiments toward the simplicity of their CDSS [56]. Furthermore, physicians are unwilling to use CDSS if it increases the time and cost [57]. Involving clinicians in the development of CDSS can improve system acceptance and adoption by ensuring that it meets the needs and preferences of users.

### Strengths and Limitations

This review was conducted rigorously and adhered to established guidelines, including the JBI methodological guidance for scoping reviews and the PRISMA-ScR statement, ensuring transparency and credibility of the review [27,28]. In addition, 2 independent reviewers (ZAT and CQYH) were involved in the complete review process, which reduced potential biases. Furthermore, a systematic search was used to ensure a comprehensive coverage of the available literature.

Owing to the heterogeneous nature of the data included in this review, statistical analysis was not feasible, even among studies with similar objectives. Therefore, a rigorous and transparent scoping review was conducted to elucidate the mechanisms of action, effectiveness, and user acceptance of the CDSS for spinal disorders, with the hope of fostering interdisciplinary understanding and collaboration.

The methodology of this scoping review did not require a formal quality assessment of the included studies, and consequently, such an evaluation was not conducted. We recognize that the quality of the literature incorporated is crucial in shaping the outcomes of this study, thus constituting a limitation to the findings. During the screening process for study inclusion, interrater reliability was not systematically evaluated, representing another acknowledged limitation of this study. However, to address potential inconsistencies in judgment, we actively engaged in discussions and sought the input of a third reviewer (BB) to reach a consensus.

The current implementation of CDSSs for spinal disorders is fragmented and inconsistent, which poses a challenge to comprehending and advancing this field. The lack of a standardized reporting structure in the reviewed studies presents a limitation in quantifying the effectiveness of the CDSS. To better understand the impact of CDSS on health care delivery and optimize its use in clinical practice, further research with standardized reporting methods is needed.

Our recommendation for future work is to focus on assessing the quality of prediction models while adhering to transparent reporting guidelines, such as the *Transparent Reporting of Multivariable Prediction Models for Individual Prognosis or Diagnosis—Systematic Reviews and Meta-Analyses* [75]. Specifically, we suggest systematically evaluating models using validated tools, such as the Checklist for Critical Appraisal and Data Extraction for Systematic Reviews of Prediction Modeling Studies to extract prognostic model studies and the Prediction Model Study Risk of Bias Assessment Tool to assess the quality of these models [76,77]. It is important to prioritize these efforts to ensure that the models are thoroughly evaluated and that their quality is properly assessed before application.

### Conclusions

Previous studies assessing CDSS effectiveness typically focused on the concordance between CDSS recommendations and health care providers’ decisions. A more favorable approach involves directly comparing CDSS suggestions with real clinical outcomes. To enhance CDSS development, future research should prioritize seamless system integration, considering end users’ requirements. In addition, investigations into external validation and impact studies are essential for a thorough evaluation of the system’s effectiveness across diverse health care settings. Emphasizing these factors will contribute to a more robust understanding of CDSS performance and its potential for broader implementation in the clinical practice for spinal disorders.

## Appendix

Multimedia Appendix 1 Search strategy for PubMed.

Multimedia Appendix 2 PRISMA ScR Checklist.

## Abbreviations

AI: artificial intelligence
CDSS: clinical decision support system
JBI: Joanna Briggs Institute
LBP: low back pain
LS-MRI: lumbar spine–magnetic resonance imaging
MRI: magnetic resonance imaging
PRISMA-ScR: Preferred Reporting Items for Systematic Reviews and Meta-analyses Extension for Scoping Reviews
