# Supplementary material for: Clinical Decision Support System Used in Spinal Disorders: Scoping Review
Source: J Med Internet Res. 2024 Mar 19;26:e53951. doi: 10.2196/53951 (PMC10988379; doi:10.2196/53951)
Supplement: Multimedia Appendix 1 [file jmir_v26i1e53951_app1.docx]

**Supplementary material**

Supplementary Table 1. Search strategy for PubMed

| Database | Search number | Search Terms | Results |
| --- | --- | --- | --- |
| PubMed | #1 | spine[Title/Abstract] OR spinal[Title/Abstract] OR vertebra*[Title/Abstract] OR intervertebral[Title/Abstract] OR cervical[Title/Abstract] OR back[Title/Abstract] OR lumbar[Title/Abstract] OR sacrum[Title/Abstract] OR coccyx[Title/Abstract] OR "cord injury"[Title/Abstract] OR "spinal stenosis"[Title/Abstract] OR radiculopathy[Title/Abstract] OR "back pain"[Title/Abstract] OR dorsalgia[Title/Abstract] OR lumbago[Title/Abstract] OR sciatica[Title/Abstract] OR (disc[Title/Abstract] AND (herniat*[Title/Abstract] OR degenerat*[Title/Abstract])) OR myelopathy[Title/Abstract] OR kyphosis[Title/Abstract] OR lordosis[Title/Abstract] OR scoliosis[Title/Abstract] OR spondylosis[Title/Abstract] OR spondylolisthesis[Title/Abstract] OR spondylitis[Title/Abstract] OR discitis[Title/Abstract] OR arachnoiditis[Title/Abstract] OR spondylarthritis[Title/Abstract] OR spondylolysis[Title/Abstract] OR whiplash[Title/Abstract] | 1,024,612 |
|  | #2 | spine [majr] OR "Spinal Diseases"[majr] OR "Spinal Cord"[majr] OR "Back Pain"[majr] OR "Spinal Cord Diseases"[majr] OR "Back Injuries"[majr] OR "Spinal Nerves"[majr] OR "Radiculopathy"[majr] | 441,857 |
|  | #3 | #1 OR #2 | 1,169,718 |
|  | #4 | ((decision*[Title/Abstract] AND (support*[Title/Abstract] OR aid[Title/Abstract])) OR “decision support system”[Title/Abstract] OR “clinical decision support”[Title/Abstract]) | 105,117 |
|  | #5 | "decision support systems, clinical"[majr] OR "decision support techniques"[majr] | 34,803 |
|  | #6 | #4 OR #5 | 131,727 |
|  | #7 | #3 AND #6 | 3,703 |
